# THE IMPACT OF INTERGENERATIONAL SOLIDARITY ON GRANDPARENT WELL-BEING

**DOI:** 10.1093/geroni/igad104.0402

**Published:** 2023-12-21

**Authors:** Rachel Scott, Danielle Nadorff

**Affiliations:** Mississippi State University, Mississippi State, Mississippi, United States; Mississippi State University, Mississippi State, Mississippi, United States

## Abstract

Bowen’s Family Systems theory provides a framework for examining how values and beliefs are transmitted between family members and the overall potential impact that transmission may have on the well-being of family members. While familial emotional closeness has been shown to predict well-being for older adults, there is still a lack of research investigating the influence of level of agreement on ideology among family members within this relation. Objective: This study examined the impact that level of agreement on religious and political values (consensual solidarity) has on the relation between emotional closeness in intergenerational relationships (affectual solidarity) and well-being in grandparents. Method: Using a sample from the Longitudinal Study of Generations, the current study examined grandparent-adult child (N = 336) and grandparent-grandchild (N = 239) dyads to assess the extent to which agreement on ideological beliefs moderates the relation between intergenerational emotional closeness and well-being in the grandparent generation.

Results: Affectual solidarity ratings among the three generations, as well as religious ideological differences between grandparents and grandchildren, were found to influence the well-being of grandparents. Additionally, model fit was found to be excellent for both moderation models between the three generations.

Discussion: These findings suggest that emotional closeness is a predominant factor in predicting well-being in grandparents that may not be as heavily influenced by the level of agreement on ideological beliefs as is often assumed.

